# Eosinophils in the blood of hematopoietic stem cell transplanted patients are activated and have different molecular marker profiles in acute and chronic graft-versus-host disease

**DOI:** 10.1002/iid3.25

**Published:** 2014-07-07

**Authors:** Julia Cromvik, Marianne Johnsson, Krista Vaht, Jan-Erik Johansson, Christine Wennerås

**Affiliations:** 1 Department of Hematology and Coagulation, University of GothenburgGöteborg, Sweden; 2 Department of Infectious Diseases, Sahlgrenska Academy, University of GothenburgGöteborg, Sweden

**Keywords:** Corticosteroids, eosinophils, flow cytometry, graft-versus-host disease, hematopoietic stem cell transplantation

## Abstract

While increased numbers of eosinophils may be detected in patients with graft-versus-host disease (GVHD) following hematopoietic stem cell transplantation, it is not known if eosinophils play a role in GVHD. The aims of this study were to determine: whether eosinophils are activated during GVHD; whether the patterns of activation are similar in acute and chronic GVHD; and the ways in which systemic corticosteroids affect eosinophils. Transplanted patients (*n* = 35) were investigated for eosinophil numbers and the expression levels of 16 eosinophilic cell surface markers using flow cytometry; all the eosinophil data were analyzed by the multivariate method OPLS-DA. Different patterns of molecule expression were observed on the eosinophils from patients with acute, chronic, and no GVHD, respectively. The molecules that provided the best discrimination between acute and chronic GVHD were: the activation marker CD9; adhesion molecules CD11c and CD18; chemokine receptor CCR3; and prostaglandin receptor CRTH2. Patients with acute or chronic GVHD who received systemic corticosteroid treatment showed down-regulation of the cell surface markers on their eosinophils, whereas corticosteroid treatment had no effect on the eosinophil phenotype in the patients without GVHD. In summary, eosinophils are activated in GVHD, display different activation profiles in acute and chronic GVHD, and are highly responsive to systemic corticosteroids.

## Introduction

Transplantation of hematopoietic stem cells isolated from the peripheral blood or bone marrow of a related or unrelated donor may be used to cure hematologic malignancies, aplastic anemia, and primary immune deficiencies. About half of all transplanted patients develop a condition called graft-versus-host disease (GVHD), which can range from mild to severe and may significantly impair the quality of life. There are at least two forms of GVHD, acute and chronic; patients may even present with features of both acute and chronic GVHD, in what is termed “overlap” GVHD. Acute GVHD (aGVHD) occurs when donor-derived T lymphocytes recognize foreign (allogeneic) antigens on the recipient's cells in tissues, which are primarily the skin, intestine, liver, and bile ducts. This T-cell-mediated inflammatory process can be further amplified through cytokine secretion, triggering of apoptosis, and other cytotoxic mechanisms [1]. The pathogenic mechanisms underlying the development of chronic GVHD (cGVHD) are less well understood but they involve immune dysregulation, incapacity to maintain central and peripheral tolerance, and the aforementioned allogeneic T-cell reactivity. Corticosteroids are currently the first line of treatment for patients with GVHD, but the options for corticosteroid-refractory GVHD are limited, making this a life-threatening condition.

The first mention of an association between eosinophils and GVHD was made in 1980, when Shulman reported that increased levels of blood eosinophils were detected in 78% of bone marrow transplant recipients with cGVHD, as compared with 12% of transplanted patients without GVHD [2]. More recent studies have documented lower rates of eosinophilia in cGVHD, in the order of 15–44% [3–5]. Eosinophils have also been shown to feature in various manifestations of cGVHD that involve the skin, muscles, eyes, and lungs [6–9]. In fact, blood eosinophilia has been proposed as a biomarker for cGVHD by the NIH Consensus Group [10]. There also appears to be an association between eosinophils and aGVHD, as eosinophilia often precedes the development of aGVHD [11–14]. Eosinophils are also seen in the tissues of patients who are afflicted with aGVHD: in a systematic study of duodenal biopsies collected from transplant recipients who had digestive symptoms, eosinophils were only detected in the patients with GVHD, and the numbers of eosinophils correlated with the severity of GVHD [12]. Eosinophils have also been found in the skin of patients with aGVHD, although their presence is not a diagnostic criterion, since there are considerably more eosinophils in the skin of patients with drug hypersensitivity reactions [15]. Intriguingly, several studies have indicated eosinophilia as a favorable prognostic factor, associated with a less-severe course of aGVHD or cGVHD, as well as improved overall survival [5,6,16–19]. Nevertheless, there is concern that eosinophil infiltration may be harmful to the organs of patients afflicted with GVHD [20].

The function of the eosinophil in GVHD has not been elucidated to date. A key question is whether it is a bystander cell or an activated cell in patients with GVHD. To the best of our knowledge, only two studies have addressed this issue. A study of the phenotypes of the blood-borne eosinophils of patients with aGVHD revealed that 5/7 patients had detectable levels of the IL-2 receptor subunit CD25, which is considered to be a marker of activation [21]. Daneshpouy et al. [12] found that the eosinophils present in intestinal biopsies from patients with GVHD expressed IL-5 and had released the granule-localized protein eosinophil peroxidase, which is also indicative of cell activation. Apart for their role in the defense against helminthic parasites, the functions of eosinophils remain elusive. Recently, it has been suggested that eosinophils can regulate adaptive immune responses [22]. For example, purified eosinophilic granule-stored proteins have been shown to inhibit lymphocyte proliferation in vitro [23], and the cells themselves can skew T-cell responses towards Th2 [24] or amplify both Th1 and Th2 types of cytokine secretion [25]. Furthermore, eosinophils are necessary for the maintenance of humoral immune memory through promotion of the long-term survival of plasma cells in the bone marrow of mice [26]. Finally, it has been proposed that thymic eosinophils participate in the regulation of adaptive immunity [27].

The aims of the present study were to determine whether blood eosinophils are activated in GVHD, and if so, whether they are differentially activated in acute and chronic GVHD, respectively. A panel of surface markers that have previously been shown to be altered in patients with other eosinophil-associated diseases were selected [28]. In addition, we evaluated how systemic treatment with corticosteroids affects the phenotypes of the blood eosinophils of patients with GVHD.

## Materials and Methods

### Patients

Adult allogeneic hematopoietic stem cell transplant recipients (*n* = 35) were recruited from the Bone Marrow Transplant Unit, Department of Hematology and Coagulation, at the Sahlgrenska University Hospital, Göteborg, Sweden. The clinical characteristics of the patients are compiled in Table1 and more detailed information is given in Table2. The clinical diagnosis and staging of GVHD were according to the criteria of the NIH Consensus Working Group [29]. The patients who received systemic treatment with corticosteroids were divided into groups. The steroid (prednisolone) was administered at the following dosages: for patients with aGVHD, median of 26 mg (range, 15–50 mg); for patients with cGVHD, median of 16 mg (range, 1.25–125 mg); and for patients without GVHD diagnosis, median of 4.6 mg (range, 1.25–20 mg). The study was approved by the Research Ethics Committee of the Medical Faculty at the University of Gothenburg and was conducted according to the provisions of the Helsinki Declaration. All patients donated blood after providing written informed consent.

**Table 1:** Characteristics of the hematopoietic stem cell transplant recipients and their episodes of graft-versus-host disease (GVHD)

	Feature	N(n)^1^	%
Patients		35	
Age, mean (range)	46 (20–68)		
Sex	Female/male	11/24	31/69
Disease	Acute myeloid leukemia	12	34
	Malignant lymphoma	10	29
	Acute lymphatic leukemia	6	17
	Chronic myeloid leukemia	2	5.7
	Other	5	14
Donor type	Unrelated	18	51
	Related	17	49
Stem cell source	Peripheral blood	30	86
	Bone marrow	5	14
Study groups^2^			
Classical acute GVHD without systemic steroids		5 (5)	
	Skin		80
	GI tract		20
Classical acute GVHD with systemic steroids		6 (7)	
	Skin		57
	GI tract		43
Chronic GVHD without systemic steroids		9 (14)	
	Moderate		64
	Mild		36
Organ engagement	Mouth, eyes, genitals, skin		
Chronic GVHD with systemic steroids		12 (17)	
	Moderate		41
	Mild		41
	Severe		18
Organ engagement	Mouth, eyes, fascia, muscles, liver, lung, skin		
Overlap GVHD without steroids		2 (3)	
Overlap GVHD with steroids		3 (3)	
Transplant recipient without GVHD without steroids		9 (15)	
Transplant recipient without GVHD with steroids		8 (14)	

1 N, number of patients; n, number of blood sampling episodes.

2 The same patient may have experienced various types of GVHD and/or no GVHD during the course of the study, and have been sampled on various occasions.

**Table 2:** Clinical features of allogeneic stem cell transplanted patients

Patient ID	Age and gender	Diagnosis	Conditioning	Donor type	HLA matching	Stem cell source
EF01	58 M	AML	RICT, Bu/Flu/ATG	SIB	10/10	PB
EF02	56 F	NHL	RICT, Tiotepa/Flu/Cy	SIB	10/10	PB
EF03	27 M	CML	MAC, Bu/Cy	SIB	10/10	PB
EF04	50 M	AML	RICT, Bu/Cy	SIB	10/10	PB
EF05	50 M	MF	RICT, Bu/Flu	SIB	10/10	PB
EF06	59 F	CLL	RICT, Bu/Flu/ATG	SIB	10/10	PB
EF07	55 M	SAA	RICT, Flu/Cy/ATG/Rit	SIB	10/10	BM
EF08	55 M	NHL	RICT, Bu/Flu	SIB	10/10	PB
EF09	50 F	KLL	RICT, Bu/Flu/ATG	MUD	9/10	PB
EF10	24 F	NHL	MAC, Cy/TBI	SIB	10/10	PB
EF11	20 M	ALL	MAC, Cy/TBI	MUD	10/10	PB
EF12	26 F	SAA	MAC, Flu/Cy/ATG/TBI	SIB	10/10	BM
EF13	59 M	CLL	RICT,Tiotepa/Flu/Cy	MUD	8/10	PB
EF14	56 M	MF	RICT, Bu/Flu/ATG	MUD	9/10	PB
EF15	57 M	CLL	RICT, Tiotepa/Flu/Cy	MUD	10/10	PB
EF16	27 M	ALL	MAC, Cy/TBI	SIB	10/10	PB
EF17	38 M	SAA	MAC, Flu/Cy/ATG/Rit/TBI	MUD	9/10	PB
EF18	34 M	ALL	MAC, Cy/TBI	MUD	10/10	BM
EF19	48 M	AML	RICT, Tiotepa/Flu/ATG, TBI	MUD	9/10	BM
EF20	68 M	MDS	RICT, Flu/Bu/ATG	SIB	10/10	PB
EF21	28 M	AML	MAC, Bu/Cy	SIB	10/10	PB
EF22	54 M	ALL	RICT, Cy/TBI/ATG	MUD	7/10	PB
EF23	29 F	AML	MAC, Bu/Cy/ATG	MUD	9/10	PB
EF24	55 F	AML	RICT, Bu/Flu/ATG	MUD	9/10	PB
EF25	51 M	AML	RICT, Bu/Flu/ATG	SIB	10/10	PB
EF26	53 M	CML	RICT, Bu/Flu	MUD	10/10	BM
EF28	56 F	AML	RICT, Flu/Cy/Alem	MUD	9/10	PB
EF30	29 M	AML	MAC, Bu/Flu	MUD	9/10	PB
EF31	41 F	ALL	MAC Cy/ATG/TBI	MUD	10/10	PB
EF32	66 M	AML	RICT, Bu/Flu/ATG	MUD	10/10	PB
EF33	40 M	MDS	MAC Bu/Flu/ATG	MUD	10/10	PB
EF35	57 F	ALL	RICT Cy/TBI	SIB	10/10	PB
EF36	60 M	CLL	RICT, ATG, TLI	SIB	10/10	PB
EF37	39 M	CLL	MAC, Bu/Cy/Rit	MUD	10/10	PB
EF38	57 M	CLL	RICT, Tiotepa/Flu/Cy	MUD	10/10	PB

M, male; F, female; AML, acute myeloid leukemia; NHL, Non-Hodgkin's lymphoma; CML, chronic myelogenous leukemia; MF, myelofibrosis; CLL, chronic lymphocytic leukemia; SAA, severe aplastic anemia; MDS, myelodysplastic syndrome; RICT, reduced intensity conditioning; MAC, myeloablative conditioning; Bu, busulfan; Flu, fludarabin; Cy, cyclophosphamide; ATG, anti-thymocyte globulin; Rit, rituximab; TBI, total body irradiation; Alem, alemtuzumab; SIB, sibling; MUD, matched unrelated donor; PB, peripheral blood; BM, bone marrow.

### Blood sample collection

For each patient, 12 mL of EDTA-anti-coagulated peripheral blood were collected on one to five occasions, that is, an individual patient can appear in the same or in different groups, before and after the diagnosis of GVHD or treatment. On average, each of the 35 patients was sampled 2.2 times, and 78 blood samples in total were collected. The blood samples were analyzed for absolute and relative eosinophil numbers using an automated cell counter (Abbott Coulter Cell-Dyn 3000; Abbott Diagnostics, Abbott Park, IL). Erythrocytes were removed from the blood samples by repeated hypotonic lysis.

### Flow cytometry

All the flow cytometry analyses were performed within 24 h of blood collection by venipuncture. Unfractionated leukocytes were incubated at 4° for 15 min in the dark with panels of fluorochrome-conjugated mouse monoclonal antibodies directed against the molecules listed in Table3. The cells were washed once with PBS after incubation. All the antibodies were purchased from BD Biosciences (San Diego, CA), except for the antibodies against CD66b (Beckman Coulter, Indianapolis, IN) and formyl peptide receptor-2 (R&D Systems, Abingdon, UK); this antibody binds to both formyl peptide receptors 1 and 2 (FPR1 and FPR2), with a preference for FPR2 (our own data, not shown). A FACSCanto II Flow Cytometer (BD Biosciences) was used to collect 100,000 events, which were analyzed using the FlowJo 7.6.5 software (Tree Star, Ashland, OR). Instead of multiple isotype controls, the Fluorescence Minus One (FMO) technique was used [30]. Eosinophils were identified within the polymorphonuclear cell gate as cells with high side scatter and low expression of CD16 (Supplementary Figure). The data are presented as median fluorescence intensity (Median-FI) values.

**Table 3:** Eosinophilic surface markers and corresponding antibodies

Molecule			Mouse monoclonal antibodies		
			
		Function	Clone	Isotype	Fluorochrome
CD9	Tetraspanin molecule	Activation marker	M-L13	IgG1, κ	PE
CD11b	Integrin α-chain Complement receptor 3	Cell adhesion molecule Forms integrin Mac-1 with CD18 Binds iC3b	D12	IgG2a, κ	PE
CD11c	Integrin α-chain Complement receptor 4	Cell adhesion molecule Forms integrin p150,95 with CD18 Binds iC3b	B-ly6	IgG1, κ	APC
CD16	Fcγ-receptor	Binds IgG with high affinity	3G8	IgG1a, κ	FITC
CD18	Integrin β2-chain	Cell adhesion molecule Partner of LFA-1, Mac-1, p150,95	6.7	IgG1a, κ	FITC
CD23	Fcε-receptor	Binds IgE with low affinity	EBVCS-5	IgG1, κ	APC
CD40	Co-receptor	Binds CD154 (CD40L) on T cells	5C3	IgG1, κ	APC
CD44	Activation marker	Adhesion molecule Binds hyaluronan	G44–26	IgG2b, κ	APC
CD49d	Very late Antigen-4 (VLA-4) α-chain	Cell adhesion molecule Bind extracellular matrix	9F10	IgG1, κ	PE
CD54	Intercellular adhesion molecule-1 (ICAM-1)	Binds CD11a/CD18	HA58	IgG1	APC
CD66b	CEACAM-8^1^	Shed during granulocyte activation	80H3	IgG1	FITC
CD66c	CEACAM-6	Activation marker	KORSA3544	IgG1	FITC
CD69	Very early activation antigen	Activation marker	FN50	IgG1, κ	APC
CD193	CCR3	Chemokine receptor Binds eotaxins	5E8	IgG2b, κ	PE
CD294	CRTH2	Chemoattractant receptor Binds PGD2	BM16	IgG2a^2^	AlexaFluor647
FPR2	Formyl Peptide Receptor-2	Chemoattractant receptor Binds *N*-formylated peptides	304405	IgG2b	APC

1 Carcinoembryonic antigen cell adhesion molecule.

2 Rat monoclonal antibody.

### Statistical analyses

We employed “Orthogonal Projection to Latent Structures-Discriminant Analysis” (OPLS-DA), a new multivariate method that is particularly suited to detect patterns in biological samples derived from relatively few patients, as the method can handle “problematic” variables that co-vary or are void of information [31]. This method is a development of principal component analysis [32], and was used to determine whether the blood eosinophils from the different patient groups (*Y*-variables: acute GVHD, chronic GVHD, overlap GVHD, no GVHD, with or without corticosteroids) had different patterns of molecules (*X*-variables: levels of 16 surface markers expressed as median fluorescent intensity values; and absolute and relative eosinophil counts). In the analysis of acute GVHD versus no GVHD, we also included degree of HLA matching between host and donor, and type of conditioning regimen (reduced intensity-conditioning or myeloablative conditioning) as *X*-variables; the purpose was to exclude that the degree of HLA mismatch and/or type of conditioning regimen contributed to any differences in eosinophil activation.

SIMCA-P+ ver. 12 software (Umetrics, Umeå, Sweden) was used for the multivariate analyses. All the data were UV-scaled, and if skewed according to the software, log-transformed before analysis. The quality of the multivariate models that were generated based on eosinophil and clinical data was defined by the explanatory capacity (R^2^Y) and stability (Q^2^Y) of each model. The variable importance (VIP) module of the software was used to remove those X-variables that made little or no contribution to the models, and an arbitrary VIP cut-off of <1.0 was applied. Variables with VIP-values >1.0 were also analyzed using the Mann–Whitney *U*-test (GraphPad Prism; GraphPad Software, La Jolla, CA) for comparisons of two groups; a *P*-value <0.05 was considered to be statistically significant.

## Results

### Blood eosinophils are activated in transplanted patients with cGVHD but not in transplanted patients without GVHD

**Figure 1:**
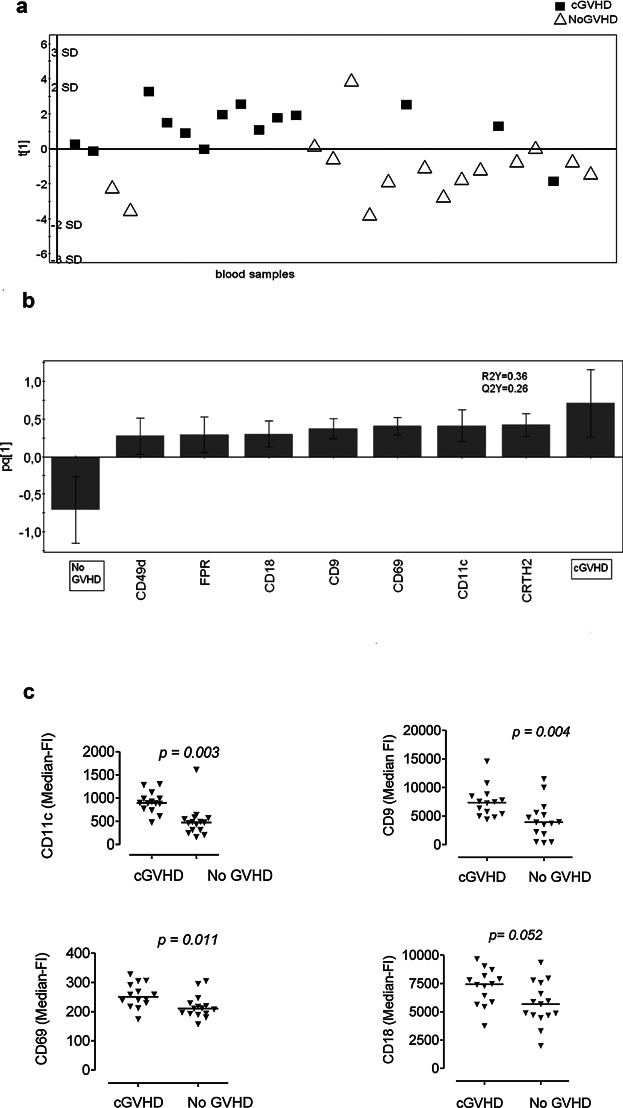
The phenotypes of the eosinophils in the blood samples of transplanted patients with chronic GVHD (cGVHD) differ from those of transplanted patients without GVHD (NoGVHD). (a) OPLS-DA score plot based on flow cytometry analysis of eosinophils in blood samples from patients with cGVHD (*n* = 9, sampled on 14 occasions) and transplanted patients without GVHD (*n* = 9; sampled on 15 occasions). Only variables with VIP-values >1.0 are included in the model. The *y*-axis indicates the degree of separation of the study groups, and the *x*-axis indicates the arbitrary order in which the samples were entered into the model. (b) Column loading plot of the eosinophilic variables. Only parameters with VIP-values >1.0 are included. X-variables that project in the same direction as the “cGVHD” column are positively associated with cGVHD, and inversely related to the “NoGVHD” column, which projects in the opposite direction. Each X-variable column displays an uncertainty bar with 95% confidence interval. (c) Mann–Whitney test of univariate statistical differences between surface marker levels on eosinophils from transplanted patients with cGVHD and transplant recipients without GVHD, respectively. Each symbol denotes one blood sample; the horizontal lines indicate the median values.

A multivariate analysis of the eosinophil data, encompassing the absolute and relative eosinophil numbers and the levels of 16 molecules expressed on the surfaces of the eosinophils derived from transplant recipients with chronic GVHD or without any form of GVHD, was conducted. A one-component model was established in which all but one of the patients with cGVHD clustered above the horizontal line corresponding to component one, and conversely, all but one of the patients without GVHD clustered below this line (Fig. 1a). This indicates that there are differences in the patterns of molecules expressed on the blood eosinophils of these two patient groups. The VIP analysis revealed that 7 of the 16 tested eosinophilic molecules contributed significantly to the variation observed between the study groups, and while all seven molecules were positively associated with cGVHD, none were positively associated with the lack of GVHD (Fig. 1b). Therefore, the eosinophils in the circulation of patients with cGVHD had higher levels of: the adhesion molecules CD11c, CD18, and CD49d; the activation markers CD9 and CD69; the prostaglandin receptor CRTH2 and the formyl peptide receptor-2 (indicated as FPR due to a certain cross-reactivity of the mAb with FPR1) than the eosinophils of transplanted patients who did not have GVHD (Fig. 1B). The results of the univariate analyses of the same molecules are shown in Figure 1c. These findings indicate that the eosinophils of patients with cGVHD are activated, whereas those of transplant recipients without GVHD are not activated.

### Blood eosinophils are activated in transplant recipients with aGVHD but not in transplant recipients without GVHD

**Figure 2:**
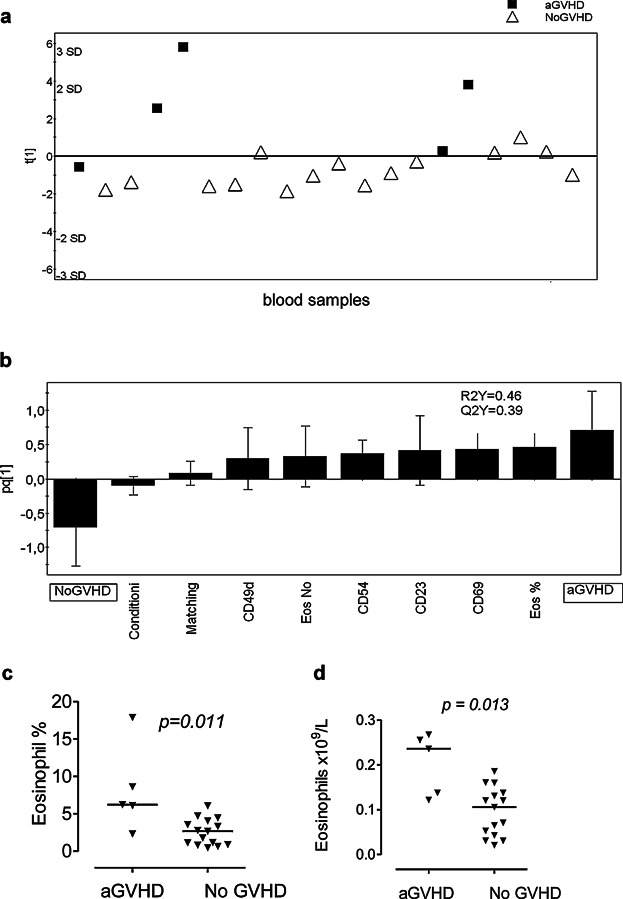
The phenotypes of the eosinophils in the blood samples of transplanted patients with acute GVHD (aGVHD) differ from those of transplanted patients without GVHD (NoGVHD). (a) OPLS-DA score plot based on flow cytometry analysis of eosinophils in blood samples from patients with aGVHD (*n* = 5) and transplanted patients without GVHD (*n* = 9; sampled on 15 occasions). Only eosinophilic variables with VIP-values >1.0 are included in the model. The two clinical variables “HLA matching” and “conditioning regimen” were also entered into the model. The *y*-axis indicates the degree of separation of the study groups, and the *x*-axis indicates the arbitrary order in which the samples were entered into the model. (b) Column loading plot of the eosinophilic variables. Only eosinophilic parameters with VIP-values >1.0 are included. X-variables columns that lie in the same direction as the “aGVHD” column are positively associated with aGVHD, and inversely related to the “NoGVHD” column. Each X-variable column displays an uncertainty bar with 95% confidence interval. (c) Mann–Whitney test of statistical differences in the absolute and relative absolute counts of eosinophils in the blood samples of transplant recipients with aGVHD or NoGVHD, respectively. Each symbol denotes one blood sample; the horizontal lines indicate the median values.

A multivariate analysis of the eosinophil patterns of patients with acute GVHD (aGVHD) versus those without GVHD (NoGVHD) was also carried out. Again, a one-component model was created in which there was clear separation of the aGVHD patients (mainly found above the horizontal line compared with non-GVHD patients who localized below the line), Figure 2a. The eosinophilic parameters that made major contributions for the segregation of the aGVHD patients from the patients without GVHD are shown in Figure 2b; these were higher absolute and relative numbers of eosinophils, raised levels of the activation marker CD69, the low-affinity IgE receptor CD23, and the adhesion molecules CD49d and CD54, as compared with the patients without GVHD. This suggests that the blood eosinophils of patients with aGVHD are activated, in contrast to the eosinophils of patients without GVHD. To exclude that the clinical variables HLA-matching and type of conditioning regimen (myeloablative or reduced-intensity) influenced the eosinophilic activation patterns and explained the differences between the study groups, we also included these as X-variables in the analysis (Fig. 2b). However, these variables had very low values in the loading scatter plot (see height of the bars) and did not contribute to the separation of the aGVHD from the NoGVHD patients (Fig. 2b). The univariate analyses confirmed that the numbers and percentages of eosinophils in the circulation were higher in the patients with aGVHD than in the patients without GVHD (Fig. 2c and d). Whereas the absolute eosinophils counts in the patients with aGVHD were within the normal range (i.e., <0.5 × 10^9^/L; Fig. 2c), the median percentage (6%) was above the reference range of 1–4% (Fig. 2d).

### Blood eosinophils have different phenotypes in acute and chronic GVHD

**Figure 3:**
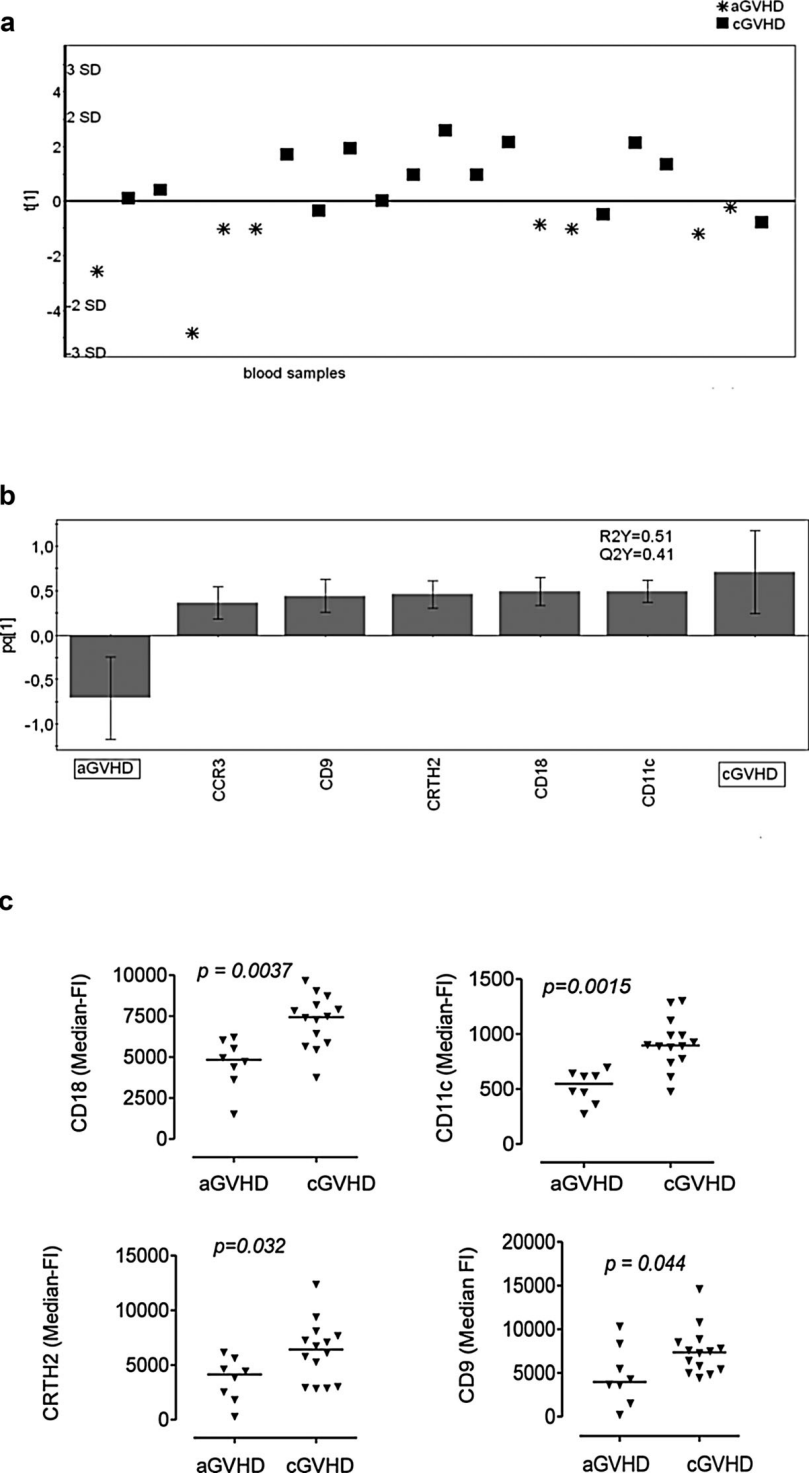
The phenotypes of the eosinophils in the blood samples of transplanted patients with acute GVHD (aGVHD) differ from those of transplanted patients with chronic GVHD (cGVHD). (a) OPLS-DA score plot based on flow cytometry analysis of eosinophils in blood samples from patients with aGVHD (*n* = 8; 5 with classical aGVHD and 3 with overlap GVHD) and transplanted patients with cGVHD (*n* = 9; sampled on 14 occasions). Only variables with VIP-values >1.0 are included in the model. The *y*-axis indicates the degree of separation of the study groups, and the *x*-axis indicates the arbitrary order in which the samples were entered into the model. (b) Column loading plot of the eosinophilic variables. Only parameters with VIP-values >1.0 are included. X-variables that lie in the same direction as the “cGVHD” column are positively associated with cGVHD and inversely related to aGVHD, since the “aGVHD” column projects in the opposite direction. Each X-variable column displays an uncertainty bar with 95% confidence interval. (c) Univariate Mann–Whitney statistical analyses of the levels of CD18, CD11c, CRTH2, and CD9 on the surfaces of blood eosinophils; data shown are median-fluorescence intensities. Each symbol denotes one blood sample, and the horizontal lines indicate median values.

The eosinophils in the blood samples of the patients with aGVHD or cGVHD seemed to be activated. However, their patterns of eosinophilic surface molecules were not identical. Consequently, we investigated whether the eosinophilic marker profiles could be used to distinguish patients with aGVHD (*n* = 5) from patients with cGVHD (*n* = 9). The three patients who had overlap GVHD, that is, with features of both acute and chronic GVHD, were reclassified as aGVHD because they clustered closer to the patients with aGVHD in the multivariate analyses (data not shown). Indeed, patients with aGVHD could be separated from patients with cGVHD based on eosinophil phenotypes (Fig. 3a). The eosinophilic variables that provided the greatest discriminatory power were: the adhesion molecules CD11c and CD18; chemokine receptor CCR3; prostaglandin receptor CRTH2; and the activation marker CD9 (Fig. 3b). The outcomes of the univariate analyses largely confirmed these findings (Fig. 3c).

### Systemic treatment with corticosteroids abrogates the activated phenotype of the eosinophils of patients with GVHD

**Figure 4:**
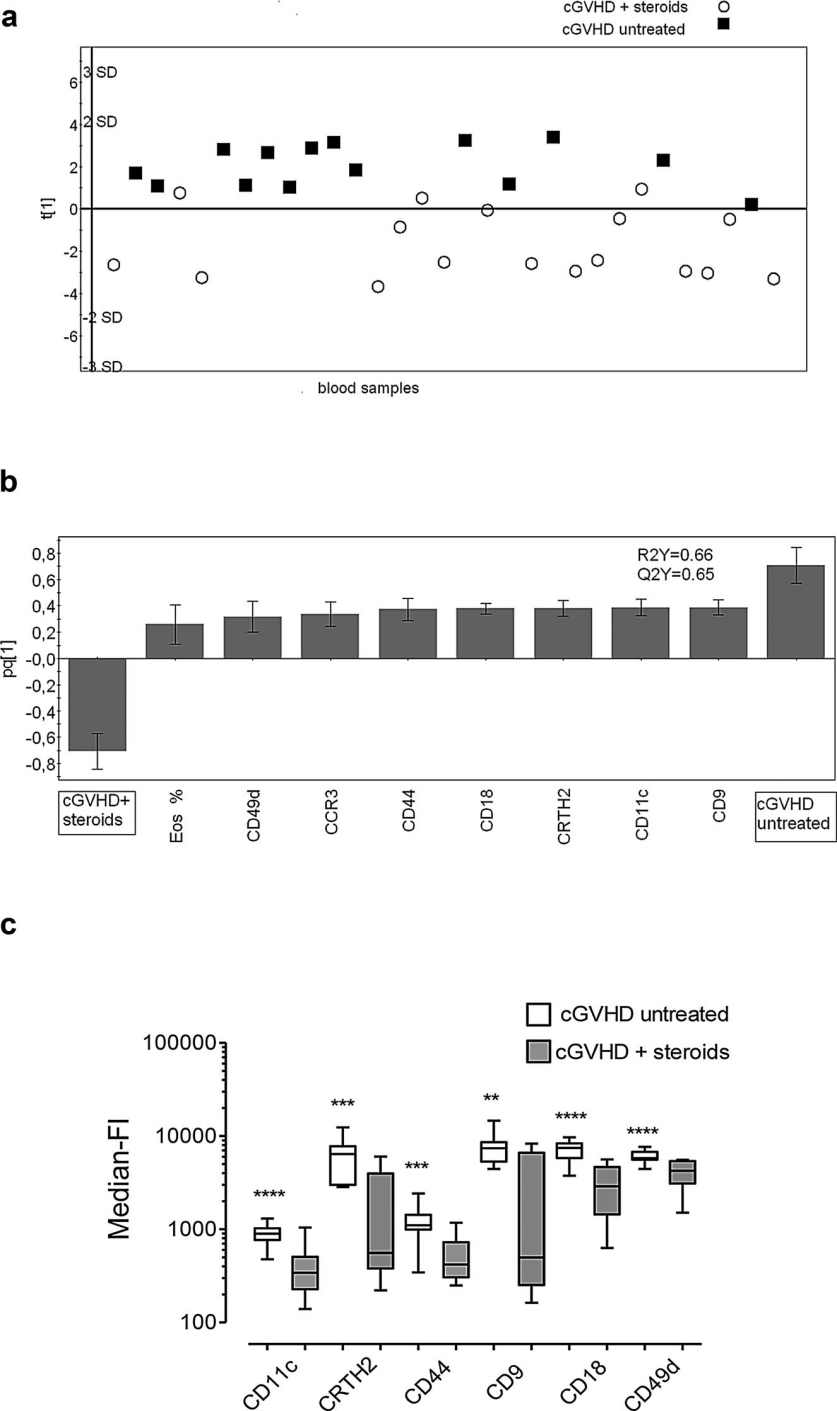
Systemic corticosteroid therapy results in global down-regulation of eosinophilic surface molecules in patients with chronic GVHD. (a) OPLS-DA score plot based on flow cytometry analysis of eosinophils in blood samples from patients with untreated cGVHD (*n* = 9; sampled on 14 occasions) and patients with cGVHD under systemic glucocorticoid treatment (*n* = 12, sampled on 17 occasions). Only variables with VIP-values >1.0 are included in the model. The *y*-axis indicates the degree of separation of the study groups, and the *x*-axis indicates the arbitrary order in which the samples were entered into the model. (b) Column loading plot of the eosinophilic variables. Only parameters with VIP-values >1.0 are included. X-variables that lie in the same direction as the “Untreated cGVHD” column are positively associated with untreated cGVHD, and inversely related to steroid-treated cGVHD, which projects in the opposite direction. Each column displays an uncertainty bar with 95% confidence interval. (c) Univariate Mann–Whitney statistical analyses of the surface levels of eosinophilic markers, shown as median-fluorescence intensities, in cGVHD patients with and without systemic corticosteroids. Data are shown as boxes with median horizontal lines and min/max whiskers. ** *P* < 0.01, *** *P* < 0.001, **** *P* < 0.0001.

To evaluate the impact of systemic treatment with corticosteroids on the eosinophilic phenotypes of patients with GVHD, multivariate analyses were performed on the eosinophilic molecule patterns in patients with or without steroid treatment. A one-component model was generated in which steroid-treated and untreated patients with cGVHD formed separate clusters (Fig. 4a). The variables that made the greatest contributions to this separation are shown in Figure 4b. It is clear that there is a positive association between the eosinophil variables and being an untreated patient with cGVHD, and a negative association between the eosinophil variables and being a patient with cGVHD who received corticosteroid treatment (Fig. 4b). Thus, the levels of molecules (CD18, CCR3, CD9, CD49d, CRTH2, CD11c, and CD44) were higher on the surfaces of eosinophils from patients with untreated cGVHD than from treated patients with cGVHD (Fig. 4b and c). The percentage of eosinophils in the circulation was significantly lower in the steroid-treated patients with cGVHD than in the untreated patients with cGVHD (Fig. 4b). Similar results were obtained for the eosinophils of patients with aGVHD, that is, systemic corticosteroids reduced the levels of eosinophilic surface markers, as compared to the levels in the untreated patients with aGVHD (data not shown).

### Systemic corticosteroids do not affect the phenotype of blood eosinophils in transplant recipients without GVHD

**Figure 5:**
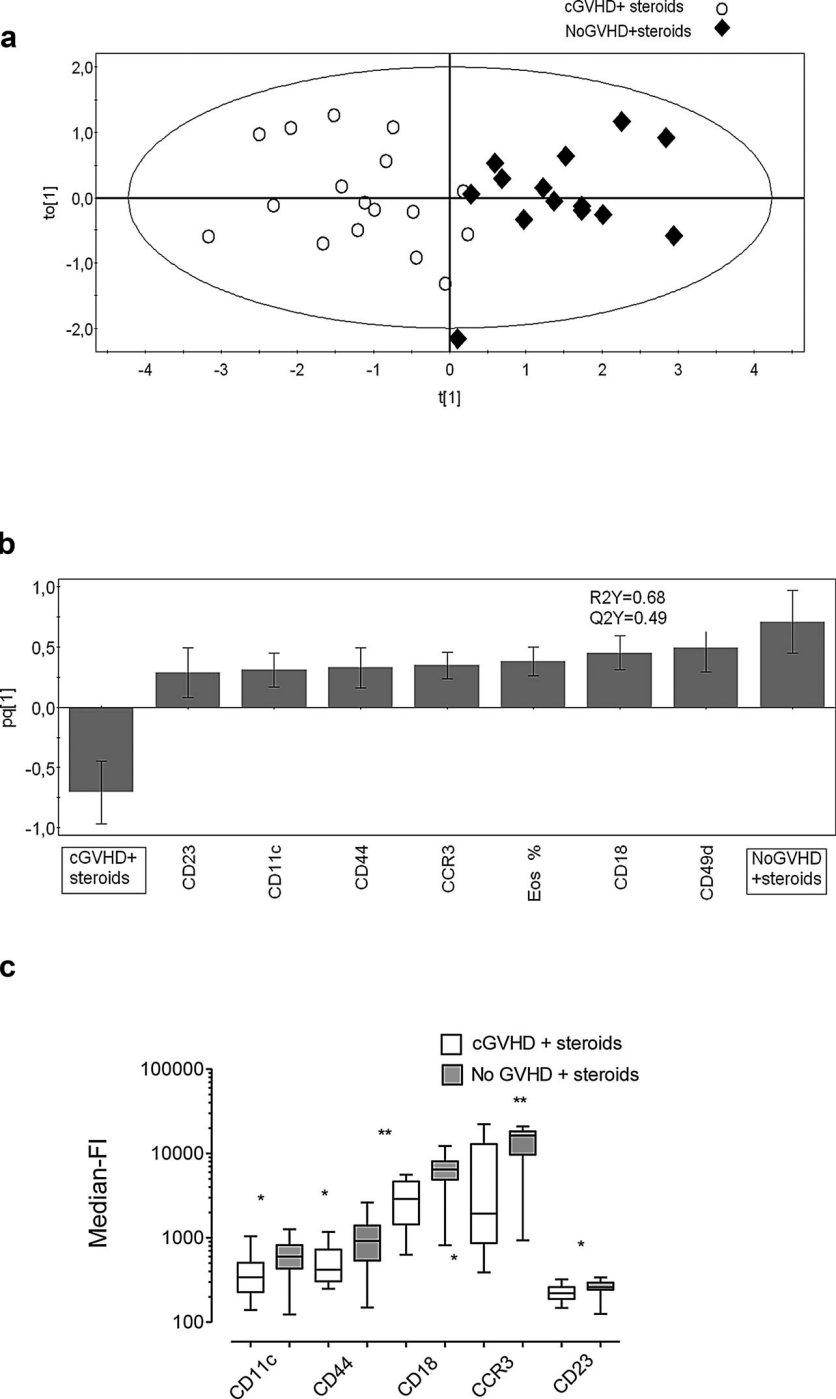
Eosinophils in blood samples from patients with chronic GVHD under systemic glucocorticoid treatment (cGVHD + steroids) have a different phenotype compared to steroid-treated transplanted patients without GVHD (NoGVHD + steroids). (a) OPLS-DA score plot based on flow cytometry analysis of eosinophils in blood samples from patients with treated cGVHD (*n* = 12; sampled on 17 occasions) and patients without GVHD under systemic glucocorticoid treatment (*n* = 8, sampled on 14 occasions). Only variables with VIP-values >1.0 are included in the model. The *y*-axis indicates the degree of separation of the study groups, and the *x*-axis indicates the arbitrary order in which the samples were entered into the model. (b) Column loading plot of the eosinophilic variables. Only parameters with VIP-values >1.0 are included. X-variables that lie in the same direction as the “No cGVHD + steroids” column are positively associated with steroid-treated GVHD patients without cGVHD, whereas parameters in the opposite direction are inversely related to steroid-treated cGVHD, which projects in the opposite direction. Each column displays an uncertainty bar with 95% confidence interval. (c) Univariate Mann–Whitney statistical analyses of the levels of surface markers on blood eosinophils from steroid-treated transplant recipients with or without cGVHD, expressed as median-fluorescence intensities (Median-FI). Data are shown as boxes with median horizontal lines and min/max whiskers. * *P* < .05, ** *P* < 0.01.

To determine whether the decreased levels of eosinophilic surface markers is a general effect of systemic corticosteroids, independent of the underlying condition, transplanted patients without GVHD who were treated with systemic corticosteroids for other reasons were investigated. In most of these cases, the indication for systemic corticosteroids was an initial suspicion of GVHD that was subsequently discarded. Multivariate analyses of the eosinophil data for steroid-treated transplant recipients with or without cGVHD generated a 2-component model, in which the two groups of patients formed separate clusters (Fig. 5a). An analysis of the phenotypes of the eosinophils of the steroid-treated patients who lacked GVHD versus those of the patients with GVHD, who received steroid treatment, showed that the former had higher levels of surface molecules (Fig. 5b). Thus, the eosinophils from steroid-treated patients without GVHD expressed higher levels of CD18, CCR3, CD49d, CD11c, CD23, and CD44 than did the eosinophils from cGVHD patients treated with systemic corticosteroids (Fig. 5b and c) In other words, systemic corticosteroids have an effect on activated cGVHD eosinophils, but have no effect (at least in terms of the markers studied) on the resting eosinophils of patients without GVHD. The same was seen for aGVHD patients, that is, systemic corticosteroids affected the phenotype of eosinophils from patients with aGVHD, but not the phenotype of eosinophils from patients without GVHD (data not shown).

## Discussion

To our knowledge, only one previous study has examined the phenotypes of eosinophils in the circulation of hematopoietic stem cell transplant recipients. Rumi et al. [21] found increased expression levels of CD9 and CD25 on the blood eosinophils from seven patients with aGVHD. Unfortunately, CD25 was not analyzed in the present study, and we did not detect increased levels of CD9 on the surfaces of the eosinophils of our patients with aGVHD. Instead, we identified an association between increased levels of CD23 and CD49d on eosinophils and aGVHD. CD23 is a low-affinity IgE receptor and a positive regulator of IgE production [33]. It has been reported that raised serum IgE levels appear after a transient increase in blood eosinophil numbers in patients with aGVHD [34]. CD23 also functions as an adhesion molecule by pairing with CD21 [33], and it may promote interactions of eosinophils with B cells and other leukocytes. Similarly, the α4-integrin CD49d, which is part of “very late antigen-4,” has been shown to enable interactions between eosinophils and lymphocytes [35]. CD49d is also important for the adherence of eosinophils to the endothelium and subsequent extravasation into tissues [36]. Finally, on the aGVHD eosinophils, the level of CD54, a molecule known to mediate the interaction of eosinophils with T cells, was increased [37].

Eosinophils are on the NIH Consensus Group's list of candidate biomarkers for the diagnosis of cGVHD [10]. Our findings strongly suggest that eosinophils are activated in cGVHD, since we show that patients with cGVHD have higher levels of the eosinophilic activation markers CD9 and CD69 [38,39] than patients without GVHD. The ligand for CD9 is as yet unidentified, although cross-linking of this molecule prolongs eosinophil survival in vitro [38]. The life-span of eosinophils is normally a few days, but it can be extended to several months when the cells take part in inflammatory reactions [40]. An appealing notion in the context of GVHD is that CD9 endows eosinophils with anti-inflammatory properties, as has been shown for macrophages [41].

The eosinophils from patients with chronic GVHD also had increased levels of CD11c and CD18, which together form the “most enigmatic” of the integrin heterodimers expressed by eosinophils [42]. CD11c binds to proteolytically degraded fibrinogen [43], which may fit with the ability of eosinophils to repair damaged tissues [44]. Alternatively, eosinophilic CD11c expression may dampen allogeneic T-cell responses, as has been shown for dendritic cells in vitro [45]. The levels of two chemoattractant receptors, CRTH2 and formyl peptide receptor-2, were increased on the eosinophils of the patients with cGVHD. CRTH2 mobilizes eosinophils from the bone marrow to the circulation, and supports the migration of eosinophils toward prostaglandin D2 and its metabolites [46]. Formyl peptide receptors bind the formyl peptides released by damaged mitochondria and may thus guide leukocytes to sites of sterile systemic inflammation [47], of which GVHD is an example.

An interesting finding was that the eosinophil phenotypes of the patients with cGVHD and aGVHD differed. Our interpretation is that eosinophils receive different activation signals from the tissues in the two forms of GVHD. This is perhaps not surprising, as it is thought that the immune pathogenetic mechanisms differ to some extent between cGVHD and aGVHD.

Corticosteroids are the first line treatment for GVHD. It is essential to understand how this therapy affects the leukocytes of patients with GVHD, as steroid-resistant GVHD is a therapeutic challenge. Corticosteroids significantly decrease eosinophil counts in the peripheral blood [48], as confirmed in the present study. A novel finding of the present study is that corticosteroids caused a general down-regulation of the cell surface markers for the activated eosinophils from patients with GVHD but not for the eosinophils of the patients without GVHD. There are very few studies that compare the effects of glucocorticosteroids on eosinophils in healthy versus inflammatory conditions. One study documented that dexamethasone treatment of eosinophil progenitors isolated from the blood of healthy and asthmatic individuals reduced the number of colony-forming units only in the asthmatics [49].

One criticism of our findings concerns the dosage of corticosteroids given to the patients without GVHD. While the dosage was lower than that administered to the patients with aGVHD, it was of the same order as that given to patients with cGVHD. Our findings reinforce the conclusion that the eosinophils in GVHD are activated and can be modulated by corticosteroids, whereas the eosinophils in the blood of transplant recipients without GVHD are not activated and therefore cannot be modulated by corticosteroids.

In summary, the results of the present study suggest that eosinophils play a role in GVHD, and have different functions in acute and chronic GVHD. However, the precise nature of what functions eosinophils have in GVHD cannot be deduced from the present study. It is tempting to speculate that eosinophils might function as immunoregulatory cells although the opposite could be true. Eosinophil phenotypes could perhaps be used as biomarkers to aid in the diagnosis of GVHD, and to predict the development of GVHD.

## SUPPORTING INFORMATION

**Figure S1.** Gating of eosinophil was performed on unfractioned leukocytes following repeated hypotonic lysis.
