# Supplementary material for: Eosinophils in the blood of hematopoietic stem cell transplanted patients are activated and have different molecular marker profiles in acute and chronic graft-versus-host disease
Source: Immun Inflamm Dis. 2014 Jul 7;2(2):99–113. doi: 10.1002/iid3.25 (PMC4217552; doi:10.1002/iid3.25)
Supplement: Supplementary file 1 — Figure S1. Gating of eosinophil was performed on unfractioned leukocytes following repeated hypotonic lysis. [file iid30002-0099-SD1.pdf]

Supplemental Data

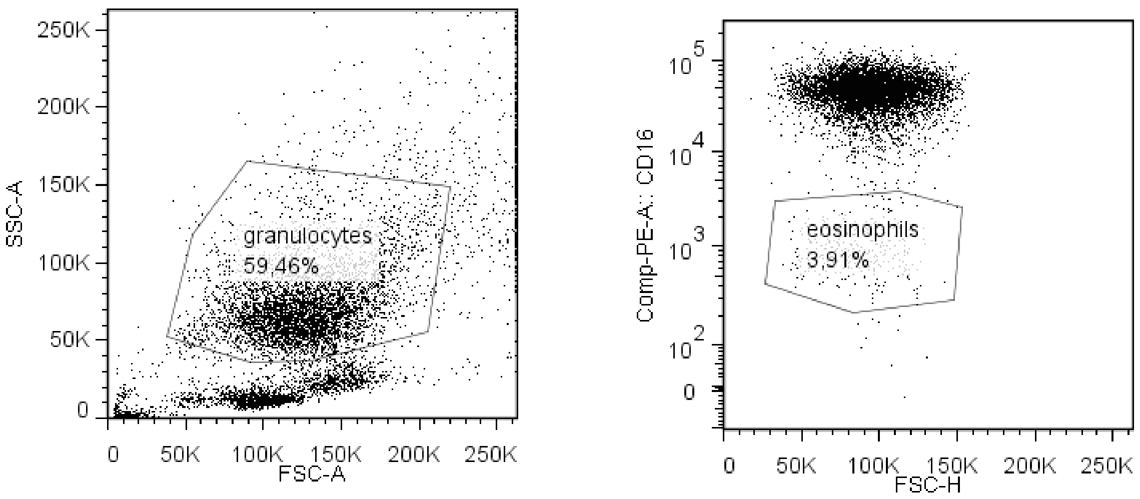

SUPPLEMENTARY FIGURE. Gating of eosinophil was performed on unfractioned leukocytes following repeated hypotonic lysis.
